# Targeted gene expression profiling predicts meningioma outcomes and radiotherapy responses

**DOI:** 10.21203/rs.3.rs-2663611/v1

**Published:** 2023-03-20

**Authors:** David Raleigh, William Chen, Abrar Choudhury, Mark Youngblood, Mei-Yin Polley, Calixto-Hope Lucas, Kanish Mirchia, Sybren Maas, Abigail Suwala, Minhee Won, James Bayley, Akdes Harmanci, Arif Harmanci, Tiemo Klisch, Minh Nguyen, Harish Vasudevan, Kathleen McCortney, Theresa Yu, Varun Bhave, Tai-Chung Lam, Jenny Pu, Gilberto Leung, Jason Chang, Haley Perlow, Joshua Palmer, Christine Haberler, Anna Berghoff, Matthias Preusser, Theodore Nicolaides, Christian Mawrin, Sameer Agnihotri, Adam Resnick, Brian Rood, Jessica Chew, Jacob Young, Lauren Boreta, Steve Braunstein, Jessica Schulte, Nicholas Butowski, Sandro Santagata, David Spetzler, Nancy Ann Oberheim Bush, Javier Villanueva-Meyer, James Chandler, David Solomon, C Rogers, Stephanie Pugh, Minesh Mehta, Penny Sneed, Mitchel Berger, Craig Horbinski, Michael McDermott, Arie Perry, Wenya Bi, Akash Patel, Felix Sahm, Stephen Magill

**Affiliations:** University of California San Francisco; UCSF; University of California, San Francisco; Northwestern University; NRG Statistics and Data Management Center; Johns Hopkins; Mayo Clinic; Leiden University Medical Center; Heidelberg University Hospital; NRG Statistics and Data Management Center; Baylor College of Medicine; Baylor College of Medicine; THE UNIV OF TX HEATH SCIENCE CTR; Baylor College of Medicine; University of California San Francisco; University of California San Francisco; Northwestern University; University of California San Francisco; Brigham and Women's Hospital; The University of Hong Kong; The University of Hong Kong; The University of Hong Kong; University of California San Francisco; The Ohio State University; The Ohios State University James Comprehensive Cancer Center; Medical University of Vienna; Medical University of Vienna; Medical University of Vienna; Caris Life Sciences; University Hospital Magdeburg; Sonia and Arthur Labatt Brain Tumour Research Centre; The Children's Hospital of Philadelphia; Center for Cancer and Immunology Research, Children’s National Research Institute; University of California San Francisco; University of California San Francisco; University of California San Francisco; Department of Radiation Oncology, University of California San Francisco, San Francisco California; University of California San Diego; University of California, San Francisco; Brigham and Women's Hospital; Caris Life Sciences International; University of California San Francisco; UCSF; Northwestern University; University of California, San Francisco; NRG Statistics and Data Management Center; NRG Oncology Statistics and Data Management Center; NRG Statistics and Data Management Center; University of California San Francisco; University of California San Francisco (UCSF); Northwestern University; Baptist Health; UCSF; Brigham and Women's Hospital; Baylor College of Medicine; University Hospital Heidelberg; Northwestern University

**Keywords:** Biomarker, brain, cancer, central nervous system, gene expression, genomics, meningioma, radiation, radiotherapy, tumor

## Abstract

**Background:**

Surgery is the mainstay of treatment for meningioma, the most common primary intracranial tumor, but improvements in meningioma risk stratification are needed and current indications for postoperative radiotherapy are controversial. Recent studies have proposed prognostic meningioma classification systems using DNA methylation profiling, copy number variants, DNA sequencing, RNA sequencing, histology, or integrated models based on multiple combined features. Targeted gene expression profiling has generated robust biomarkers integrating multiple molecular features for other cancers, but is understudied for meningiomas.

**Methods:**

Targeted gene expression profiling was performed on 173 meningiomas and an optimized gene expression biomarker (34 genes) and risk score (0 to 1) was developed to predict clinical outcomes. Clinical and analytical validation was performed on independent meningiomas from 12 institutions across 3 continents (N = 1856), including 103 meningiomas from a prospective clinical trial. Gene expression biomarker performance was compared to 9 other classification systems.

**Results:**

The gene expression biomarker improved discrimination of postoperative meningioma outcomes compared to all other classification systems tested in the independent clinical validation cohort for local recurrence (5-year area under the curve [AUC] 0.81) and overall survival (5-year AUC 0.80). The increase in area under the curve compared to the current standard of care, World Health Organization 2021 grade, was 0.11 for local recurrence (95% confidence interval [CI] 0.07–0.17, P < 0.001). The gene expression biomarker identified meningiomas benefiting from postoperative radiotherapy (hazard ratio 0.54, 95% CI 0.37–0.78, P = 0.0001) and re-classified up to 52.0% meningiomas compared to conventional clinical criteria, suggesting postoperative management could be refined for 29.8% of patients.

**Conclusions:**

A targeted gene expression biomarker improves discrimination of meningioma outcomes compared to recent classification systems and predicts postoperative radiotherapy responses.

## Introduction

Meningiomas comprise 39.7% of primary intracranial tumors and are the only brain tumors that are more common in women, Black, and elderly patients, who are underrepresented in brain tumor clinical trials^1,2^. Meningioma treatments are largely restricted to surgery and radiotherapy, and systemic therapies remain ineffective or experimental^3,4^. Historically, the World Health Organization (WHO) has graded meningiomas according to histological features such as mitotic count^5^. Most WHO grade 1 meningiomas can be effectively treated with surgery or radiotherapy, but many WHO grade 2 or grade 3 meningiomas (which account for 20–30% of cases^1^) are resistant to treatment and cause significant neurological morbidity and mortality^3^. Moreover, some WHO grade 1 meningiomas develop recurrences that cannot be predicted from histological features, and some WHO grade 2 or grade 3 meningiomas are unexpectedly well controlled with surgery and radiotherapy. In recognition of the controversies surrounding meningioma risk stratification and treatment, the NRG BN-003 and EORTC 1308 Phase III clinical trials randomize patients with primary WHO grade 2 meningiomas to postoperative surveillance or postoperative radiotherapy after gross total resection^6^. The only multicenter prospective studies of meningioma radiotherapy that have reported data are RTOG 0539 and EORTC 22042, and these Phase II clinical trials provide safety and non-randomized outcome data based on clinical criteria that do not predict radiotherapy responses in many retrospective series^7-10^. Thus, there are unmet needs for improved risk stratification and prediction of postoperative radiotherapy responses for patients with meningiomas, the most common primary intracranial tumors.

In 2021, the WHO revised meningioma grading criteria to incorporate rare hotspot *TERT* promoter mutations and homozygous deletion of *CDKN2A/B* alongside traditional histological features^11^. The WHO 2021 update reflects a growing understanding of the molecular landscape of meningiomas from diverse bioinformatic studies. DNA sequencing^12-15^, copy number variant (CNV) analyses^16-18^, RNA sequencing^19,20^, or DNA methylation profiling^21-24^ have been used to classify meningiomas based on recurring somatic short variants^12-15^, chromosome gains and losses^16-18^, differentially expressed genes^19,20^, or DNA methylation probes^23^, families^24^, groups^22^, or subgroups^21^. Integrated systems have been proposed based on (1) CNVs, *CDKN2A/B* status, and histological features (integrated grade)^16^, (2) CNVs, DNA methylation families, and histological features (integrated score)^17^, or (3) CNVs, DNA methylation profiling, RNA sequencing, and DNA sequencing which reveal biological groups and subgroups of meningiomas that are concordant with results from DNA methylation profiling alone^18,21,22^. It is unknown which of these diverse classification system(s) may optimize risk stratification or predict postoperative radiotherapy responses for patients with meningiomas.

Knowledge of the biological pathways underlying breast, prostate, and other cancers has generated robust targeted gene expression biomarkers that are recommended for risk stratification and prediction of treatment response by the National Comprehensive Cancer Network (NCCN)^25-30^. A small pilot study suggested that targeted gene expression profiling may be useful for meningioma risk stratification^31^, but an optimized gene expression biomarker, as well as the analytical validity, clinical validity, generalizability, and potential impact of this approach on postoperative meningioma management were unknown.

Here we use known biological pathways underlying meningiomas from bioinformatic studies^11,12,21,22,31,13-20^ to develop a 34-gene expression biomarker predicting clinical outcomes in a single-institution discovery cohort. We perform clinical and analytical validation of the gene expression biomarker using independent meningiomas from a large multicenter retrospective cohort representing North America, Asia, and Europe, and compare the performance of the gene expression biomarker across contemporary meningioma classification systems and clinical contexts. We provide investigator-blinded, independent validation of the gene expression biomarker using a multicenter prospective cohort of meningiomas from patients enrolled on RTOG 0539 who represent the social and geographical landscape of the United States. In sum, our results reveal the gene expression biomarker provides additional information for meningioma outcomes compared to recent classification systems, including prediction of postoperative radiotherapy responses.

## Methods

### Study design

**Figure 1:**
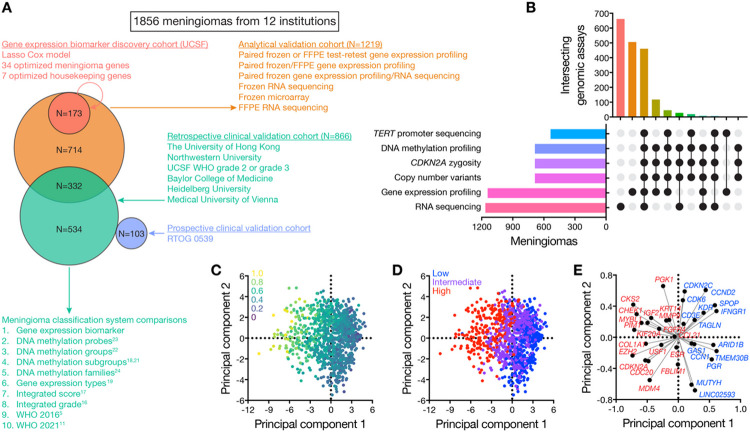
Study design and gene expression biomarker characteristics. Panel A shows the study design and numbers of meningiomas used for gene expression biomarker development, analytical validation (Figure S4, Table S4), clinical validation, and comparison across classification systems. See Supplemental Methods, Table 1, and Tables S1-S9 for additional details. Superscript numbers correspond to manuscripts reporting comparator meningioma classification systems in the References. Panel B shows an upset plot of 4898 genomic assays (horizontal) performed across 1856 unique meningiomas (vertical) to define and compare molecular classification systems in this study. Panels C and D show the distribution of continuous or discrete gene expression risk scores in principal component space. Dots represents individual meningiomas from the multicenter retrospective and prospective clinical validation cohorts (N=969). Panel E shows loading scores for the 34 genes comprising the gene expression biomarker. A simplified color scheme shows genes associated with higher risk in red and genes associated with lower risk in blue in the first 2 principal components.

A discovery cohort comprised of 173 retrospective meningiomas with well-annotated clinical follow up data from a single institution was used to identify and optimize a 34-gene expression biomarker and risk score (Figures 1A, S1 and Tables 1, S1-S3). The performance of the gene expression biomarker was validated in 3 cohorts. First, the analytical validity of the gene expression biomarker was tested in a retrospective analytical validation cohort comprised of 1219 meningiomas from 8 international institutions, some of which had sparse or absent clinical follow up data (Figure 1A and Table S4). Meningiomas from the discovery cohort, which had matched RNA sequencing, were also used for analytical validation of orthogonal approaches for gene expression quantification (Figure 1A and Table S4). Second, the clinical validity and performance of the gene expression biomarker in comparison to other meningioma classification systems were tested in an independent retrospective clinical validation cohort comprised of 866 meningiomas with well-annotated clinical follow up data from 6 international institutions (Figure 1A and Tables 1, S5-S9), some of which were also used for analytical validation (Table S4). There was no overlap among meningiomas used to identify and optimize the gene expression biomarker in the discovery cohort (Table S2) and meningiomas used for clinical validation (Tables S4-S9). Concordance index (c-index), log-rank test, Brier error score, time-dependent area under the receiver operant curve (AUC), delta-AUC, the Kaplan Meier method, multivariate analysis (Tables S10, S11), and propensity matching (Table S12) were used to compare gene expression biomarker performance across contemporary molecular and histological classification systems and clinical contexts. Third, a prospectively collected cohort of 103 meningiomas from patients enrolled on RTOG 0539 were used for investigator-blinded, independent clinical validation (Figure 1A and Tables 1, S13, S14). In total, 4898 genomic assays were performed and analyzed across 1856 unique meningiomas to define and compare molecular classification systems (Figure 1B). Details on data collection, tissue and nucleic acid processing, genomic assays, pathology review, imaging review, statistical analyses, and code and data availability are reported in the Supplemental Methods.

### Targeted gene expression analysis

Targeted gene expression profiling was performed using a hybridization and barcode-based panel with internal negative and spike-in positive controls^32^ (Supplemental Methods). Positive-control normalized gene counts were standardized by normalization to the geometric mean count of 7 meningioma-specific housekeeping genes (Table S3). Log_2_ transformed gene expression values were used for all subsequent analyses. Meningioma related genes of interest (Table S1) were selected based on prognostic or biological significance in the literature^11,12,21,22,31,13-20^ (Supplemental Methods), and feature selection was performed using a Lasso regularized Cox regression model with the c-index of local freedom from recurrence (LFFR) in the discovery cohort as the target endpoint (Table S2). An optimized set of 34 genes was identified within 1 standard error of the model achieving maximal c-index (Figure S1A and Table S3), resulting in a highly discriminatory set of linearly rescaled risk scores between 0 and 1 (Figures 1C, S2). To further reduce over-fitting and to facilitate re-calibration of the model for data derived from frozen or formalin-fixed and paraffin embedded (FFPE) meningiomas, or for data derived from orthogonal approaches for gene expression quantification such as RNA sequencing, bootstrap aggregation was used to train 500 ridge-regression sub-models using normalized and log_2_-transformed gene counts as input and discovery cohort risk scores as target variables^33^.

Gene expression risk score cutoffs for Kaplan Meier analyses were determined using a nested procedure (Figure 1D). An initial cutoff was determined in the discovery cohort using the maximally selected rank statistic. The subsets above and below this threshold were again split by maximally selected rank statistic. The lowest risk score group was considered low risk (LFFR cutoff £0.3760769, overall survival [OS] cutoff £0.4206913), and the highest risk score group was considered high risk (LFFR cutoff >0.5651741, OS cutoff >0.6453035). The intervening risk score groups were combined as intermediate risk (LFFR cutoff (0.3760769, 0.5651741], OS cutoff (0.4206913, 0.6453035]). All model training, calibration, and cutoff determination was performed in the discovery cohort (N=173).

### Reproduction of molecular classification systems in validation cohort meningiomas

Assignment of validation cohort meningiomas to DNA methylation groups^22^ or DNA methylation subgroups^21^ (WCC, AC, CHGL, HNV, STM, DRR), DNA methylation families^24^ or integrated score^17^ (SLNM, FS), or gene expression types^19^ (JCB, ASH, AH, TK, AJP) was performed independently by investigators who developed each of these classification systems. Integrated grade^16^ was assigned using CNVs derived from DNA methylation profiles and histological features under supervision of investigators who developed this classification system (SS, WLB). DNA methylation probe risk scores were estimated by training a Lasso regularized Cox regression model with LFFR as the endpoint in the discovery cohort using β-values of 283 unfavorable CpG loci^23^. The resulting continuous risk score was converted into low, intermediate, and high risk groups using the same nested procedure described for the gene expression risk score above. All meningioma classification system assignments were performed by investigators who were blinded to clinical outcomes and other molecular characteristics of the meningiomas included in this study (Figure 1A).

## Results

### Gene expression biomarker development and optimization

Targeted gene expression profiling of 173 meningiomas in the discovery cohort (Tables 1, S1, S2) resulted in a 34-gene expression biomarker and continuous risk score between 0 and 1 that was converted into discrete low, intermediate, and high risk groups for Kaplan Meier analyses (Figs. 1C-E, S1 and Table S3). The gene expression biomarker was well distributed across intracranial meningioma locations and recurring somatic short variants, and was prognostic for LFFR and OS (Figures S2, S3). The gene expression biomarker model, risk score, and cutoffs were locked and applied without alteration to multicenter retrospective and prospective validation cohorts from 12 institutions (Tables 1, S4-S9).

### Gene expression biomarker analytical validation

Analytical validity, including reproducibility over time and across laboratories, frozen and FFPE meningiomas, and different approaches for gene expression quantification, was established using the multicenter analytical validation cohort (N = 1219 meningiomas, 8 institutions, Figs. 1 A, S4 and Table S4). Test-retest conditions, different centers, and paired frozen/FFPE meningiomas generated concordant barcode hybridization gene expression risk scores (Figures S4A, S4B) that were tractable and discriminatory for meningioma outcomes when RNA sequencing or microarray approaches were used to assess the 34-gene signature (Figures S4C-S4G).

### Gene expression biomarker clinical validation and classification system comparisons

**Figure 2:**
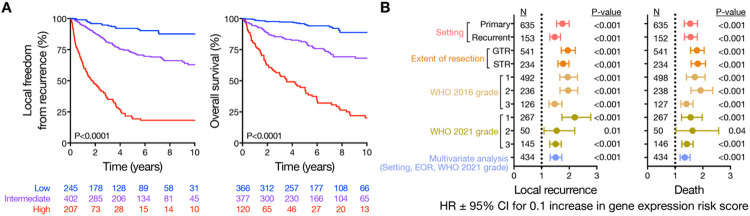
The gene expression biomarker improves discrimination of meningioma outcomes. Panel A shows Kaplan Meier curves for local freedom from recurrence (LFFR) or overall survival (OS) in the multicenter retrospective clinical validation cohort stratified by gene expression risk score (N=866 meningiomas, 6 institutions, N=854 with LFFR data, N=863 with OS data). Low, intermediate, and high gene expression risk scores were associated with 5-year LFFR of 92.2% (95% CI, 88.3-96.2%), 72.6% (95% CI 67.8-77.8%), and 19.4% (95% CI 13.5-28.0%), and 5-year OS of 95.3% (95% CI 92.9-97.8%), 83.3% (95% CI 79.3-87.5%), and 44.3% (95% CI 35.6-55.1%), respectively. Panel B shows a forest plot of hazard ratios (HR) with 95% confidence intervals (CI) for local recurrence or death in the multicenter retrospective clinical validation cohort for each 0.1 increase in gene expression risk score across meningioma settings (primary or recurrent), extent of resection (EOR), WHO grades, or multivariate analysis.

In the multicenter retrospective clinical validation cohort (N = 866 meningiomas, 6 institutions, N = 572 frozen, N = 294 FFPE, Fig. 1A and Tables S4-S9), the gene expression biomarker achieved a c-index of 0.78 for LFFR and 0.78 for OS (Figure S5). The gene expression biomarker delineated clinically meaningful low, intermediate, or high risk groups with 5-year LFFR of 92.2% (95% confidence interval [CI], 88.3–96.2%), 72.6% (95% CI 67.8–77.8%), and 19.4% (95% CI 13.5–28.0%), respectively (Fig. 2A), and remained well-calibrated in meningiomas from individual clinical validation institutions (Figure S5A). The gene expression biomarker was prognostic for LFFR and OS among meningiomas presenting in primary or recurrent settings, after gross total resection (GTR) or subtotal resection (STR), across WHO grades using histological (WHO 2016)^5^ or histological and molecular criteria (WHO 2021)^11^, and remained independently prognostic on multivariate analysis incorporating meningioma setting (primary or recurrent), extent of resection, and WHO grade (Figs. 2B, S5B and Tables S10, S11). The gene expression biomarker was prognostic for LFFR and OS within strata from other meningioma molecular classification systems based on DNA methylation probes^23^, groups^22^, subgroups^18,21^, or families^24^, or gene expression types^19^, integrated score^17^, or integrated grade^16^ (Figures S5C), and remained independently prognostic on multivariate analyses incorporating each of the 9 other meningioma classification systems (Tables S10, S11).

**Figure 3:**
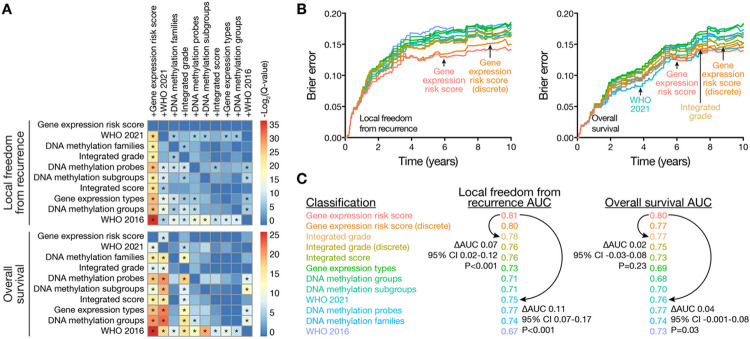
Gene expression biomarker comparisons to other meningioma classification systems. Panel A shows a heatmap of −Log_2_-transformed P-values with false-discovery-rate (FDR) correction (Q-values) for pairwise likelihood-ratio tests^34^ of improvements in Cox regression models for local freedom from recurrence (LFFR) or overall survival (OS). Meningioma classification systems in columns (e.g. +Gene expression risk score) were combined with meningioma classification systems in rows. The performance of combined models was assessed using 290 consecutive meningiomas from The University of Hong Kong validation cohort with available data to define all 10 meningioma classification systems tested. Asterixis denote Benjamini-Hochberg corrected Q<0.05. Combination with the gene expression risk score improved all other models tested for both LFFR and OS (first column). Conversely, no models improved the gene expression risk score for LFFR (first row, top heatmap), and only WHO 2021 grade provided improvement for OS (first row, bottom heatmap). These findings were additionally validated using multivariate analyses (Tables S10, S11) and Kaplan Meier analyses (Figures S5, S6). Panel B shows Brier error curves over time for LFFR or OS in the same retrospective validation cohort as Panel A. The gene expression biomarker achieved the lowest Brier error score over time for LFFR across meningioma classification systems and had an error score that was comparable to WHO 2021 grade and integrated grade over time for OS. Panel C shows 5-year time dependent area under the receiver operating characteristic (AUC) for all meningioma classification systems tested. AUC values reflect the performance of each system in all multicenter retrospective clinical validation cohort meningiomas (N=866 meningiomas, 6 institutions) with available data to define each system tested (Tables S4, S10, S11). Pairwise comparisons were performed for select systems using bootstrap delta-AUC. The gene expression biomarker achieved the highest 5-year AUC for LFFR and OS across meningioma classification systems, with a delta-AUC for LFFR of +0.07 (95% CI 0.02-0.12, P<0.001) compared to the next best performing system (integrated grade), and a delta-AUC of +0.11 for LFFR (95% CI 0.07-0.17, P<0.001) and +0.04 for OS (95% CI −0.001-0.08, P=0.03) compared to the current standard of care (WHO 2021 grade). As was the case for AUC calculations, the number of meningiomas included in each delta-AUC comparison varied depending on the number of meningiomas in the multicenter retrospective clinical validation cohort with available data to define the systems tested in each comparison (Table S4, S10, S11).

**Figure 4:**
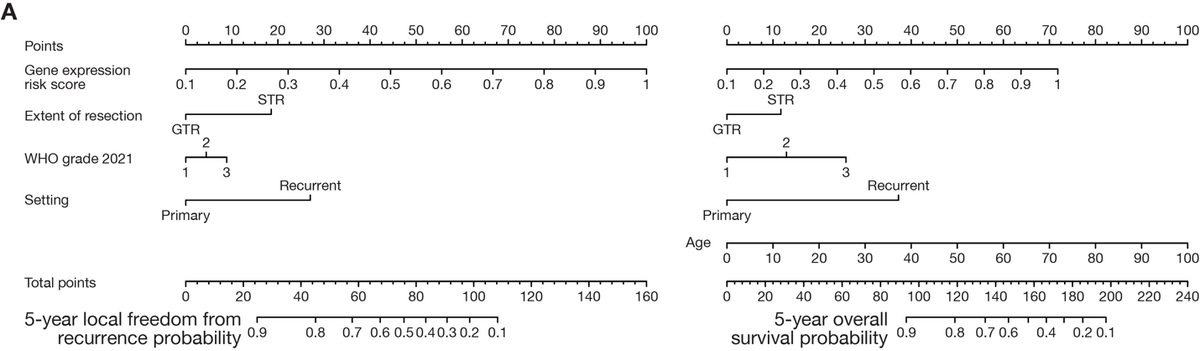
Gene expression biomarker nomograms for meningioma outcomes. Nomograms are shown for prediction of 5-year local freedom from recurrence (left) or overall survival (right) based on gene expression risk score, setting (primary or recurrent), extent of resection, and WHO 2021 grade using data from the multicenter retrospective clinical validation cohort. Similar nomograms based on WHO 2016 grade are available in Figure S7.

Comparison across meningioma classification systems based on molecular^18,19,21-24^, molecular and histological^16,17^, or WHO criteria^5,11^ using pairwise model combinations^34^ revealed the gene expression biomarker provided additional prognostic information for LFFR and OS in combination with each of the 9 other systems tested (Figs. 3A, S6A). No other meningioma classification system provided additional prognostic information for LFFR in combination with the gene expression biomarker (Figs. 3A, S6B, S6C), and only WHO 2021 grade provided additional prognostic information for OS (Fig. 3A). The gene expression biomarker achieved the lowest Brier error score over time for LFFR across meningioma classification systems, and had an error score that was comparable to WHO 2021 grade and integrated grade over time for OS (Fig. 3B). The gene expression biomarker achieved the highest 5-year AUC for LFFR (0.81) and OS (0.80) across meningioma classification systems, with a delta-AUC for LFFR of + 0.07 (95% CI 0.02–0.12, P < 0.001) compared to the next best performing system (integrated grade), and a delta-AUC for LFFR of + 0.11 (95% CI 0.07–0.17, P < 0.001) compared to the current standard of care (WHO 2021 grade) (Fig. 3C). To translate these findings into clinical practice, nomograms were generated for prediction of 5-year LFFR or OS based on meningioma gene expression risk score, setting (primary or recurrent), extent of resection, and WHO grade (Figs. 4, S7).

### Gene expression biomarker prediction of radiotherapy responses

**Figure 5:**
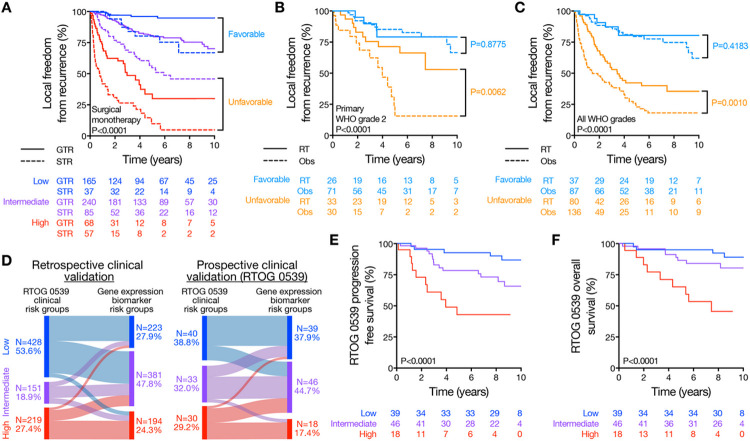
The gene expression biomarker predicts meningioma radiotherapy responses. Panel A shows Kaplan Meier curves for local freedom from recurrence (LFFR) for meningiomas in the multicenter retrospective clinical validation cohort that were treated with surgical monotherapy, stratified by extent of resection and the gene expression risk score. 5-year LFFR was 96.1% for gene expression low risk meningiomas with GTR, 80.3% for gene expression low risk with STR, 80.5% for gene expression intermediate risk with GTR, 54.9% for gene expression intermediate risk with STR, 30.0% for gene expression high risk with GTR, and 9.8% for gene expression high risk with STR. Meningiomas were grouped as favorable (N=442) or unfavorable (N=210) as shown if they had >80% or <80% 5-year LFFR, respectively, for subsequent analyses. Panel B shows Kaplan Meier curves for LFFR of favorable versus unfavorable primary WHO grade 2 meningiomas in the multicenter retrospective clinical validation cohort that received postoperative radiotherapy (RT) or underwent postoperative observation (Obs). Unfavorable primary WHO grade 2 meningiomas benefitted from postoperative radiotherapy (HR 0.33, 95% CI 0.14-0.76, P=0.009), while favorable primary WHO grade 2 meningiomas did not (P=0.88). Panel C shows Kaplan Meier curves for LFFR of favorable versus unfavorable propensity matched meningiomas in the multicenter retrospective clinical validation cohort that received postoperative radiotherapy or underwent postoperative observation. Cases were first stratified by favorable versus unfavorable criteria, and then matched based on gene expression risk score, extent of resection, and WHO grade (Table S12). Unfavorable propensity matched meningiomas benefited from postoperative radiotherapy (HR 0.54,95% CI 0.37-0.78, P=0.0001), while favorable propensity matched meningiomas did not (P=0.42). Panel D shows a Sankey plot of RTOG 0539 clinical risk groups versus gene expression biomarker risk groups in the multicenter retrospective clinical validation cohort (left) or the multicenter prospective clinical validation cohort from RTOG 0539 itself (right). Compared to clinical risk groups used for postoperative radiotherapy stratification in RTOG 0539, the gene expression biomarker re-classified 52.0% (N=416, Table S15) of retrospective validation cohort meningiomas, and 39.8% (N=41, Table S15) of RTOG 0539 meningiomas. Reclassified meningiomas were better stratified by gene expression risk (Figure S8D). Panel E shows Kaplan Meier curves for progression free survival (PFS) of patients enrolled on RTOG 0539, stratified by meningioma gene expression risk score. 5-year PFS was 92.0%, 76.5%, and 38.6% for gene expression low, intermediate, and high risk groups, respectively. Panel F shows Kaplan Meier curves for overall survival (OS) of patients enrolled on RTOG 0539, stratified by meningioma gene expression risk score. 5-year OS was 94.7%, 85.7%, and 63.0% for gene expression low, intermediate, and high risk groups, respectively.

To incorporate the gene expression biomarker into a clinical framework consistent with contemporary NCCN and European Association of Neuro-Oncology (EANO) guidelines^4,35^, meningiomas treated with surgical monotherapy in the multicenter retrospective clinical validation cohort were stratified by extent of resection and gene expression risk score, resulting in a range of clinical subgroups spanning the spectrum of recurrence risk from 5-year LFFR of 96.1% for gene expression low risk meningiomas with GTR, to 9.8% for gene expression high risk meningiomas with STR (Fig. 5A). Based on these combined biomarker/surgical strata, favorable and unfavorable meningiomas were distinguished using (1) gene expression low risk with any resection, or gene expression intermediate risk with GTR (favorable), versus (2) gene expression intermediate risk with STR, or gene expression high risk with any resection (unfavorable) (Fig. 5A).

In clinical practice, meningiomas with unfavorable histological features or STR are often treated with postoperative radiotherapy based on retrospective data^4,6,35^. NRG BN-003 and EORTC 1308 represent important prospective studies of radiotherapy for meningioma, but these trials were initiated before the development of biomarkers for risk stratification, and they do not incorporate biomarkers potentially elucidating postoperative radiotherapy responses, as defined by a reduced risk of recurrence. In the multicenter retrospective clinical validation cohort, the gene expression biomarker remained prognostic for primary meningioma outcomes among patients receiving fractionated postoperative radiotherapy (Figure S8A), and among patients with primary WHO grade 2 meningiomas with GTR who may have been eligible for NRG BN-003 and EORTC 1308 (Figure S8B). However, in the absence of biomarker stratification, primary WHO grade 2 meningiomas with GTR did not benefit from postoperative radiotherapy in the multicenter retrospective clinical validation cohort (Figure S8C). Thus, to determine if the gene expression biomarker could predict meningioma radiotherapy responses, primary WHO grade 2 meningiomas were stratified based on favorable versus unfavorable biomarker/surgical criteria (Fig. 5A), revealing that unfavorable primary WHO grade 2 meningiomas benefitted from postoperative radiotherapy (HR 0.33, 95% CI 0.14–0.76, P = 0.009) but favorable primary WHO grade 2 meningiomas did not (P = 0.88) (Fig. 5B). Applying the same biomarker/surgical strata across all WHO grades in the multicenter retrospective clinical validation cohort with propensity matching based on gene expression risk score, extent of resection, and WHO grade revealed that unfavorable meningiomas benefitted from postoperative radiotherapy (HR 0.54, 95% CI 0.37–0.78, P = 0.0001) but favorable meningiomas did not (P = 0.42) (Fig. 5C and Table S12).

RTOG 0539 was a Phase II multicenter prospective trial that enrolled patients with meningiomas from 78 institutions into 3 clinical risk groups: (1) low clinical risk comprised of primary WHO grade 1 meningiomas after any resection, (2) intermediate clinical risk comprised of recurrent WHO grade 1 meningiomas after any resection, or primary WHO grade 2 meningiomas after GTR, and (3) high clinical risk comprised of WHO grade 3 meningiomas after any resection, recurrent WHO grade 2 meningiomas after any resection, and primary WHO grade 2 meningiomas after STR. Intermediate and high clinical risk patients enrolled on RTOG 0539 received postoperative radiotherapy^8,9^, and low clinical risk patients underwent postoperative surveillance^7^. To determine how the gene expression biomarker could potentially refine postoperative management, meningiomas in the multicenter retrospective clinical validation cohort were assigned to RTOG 0539 clinical risk groups and compared across assignments to gene expression biomarker risk groups. The gene expression biomarker improved discrimination of meningioma outcomes across clinical groups used for postoperative radiotherapy stratification in RTOG 0539 (Figure S8D) and re-classified 52.0% (Table S15) of meningiomas compared to clinical criteria, including downstaging 21.3% of intermediate clinical risk patients who would have received postoperative radiotherapy on RTOG 0539 (Fig. 5D). Using favorable versus unfavorable biomarker/surgical strata that predict radiotherapy responses (Figs. 5A-5C), postoperative management could have been refined for 29.8% of patients in the multicenter retrospective clinical validation cohort compared to clinical criteria from RTOG 0539.

Investigator-blinded, independent validation of the gene expression biomarker was performed using meningiomas and clinical data that were prospectively collected from patients enrolled on RTOG 0539 itself (N = 103, Tables 1, S13). In comparison to clinical risk groups used to allocate patients to postoperative radiotherapy or postoperative surveillance on this study, the gene expression biomarker re-classified 39.8% of meningiomas from RTOG 0539 (Fig. 5D, Table S15), including downstaging 30.3% of intermediate clinical risk patients who received postoperative radiotherapy (Fig. 5D, Table S15). The gene expression biomarker was prognostic for progression free survival (PFS) and OS in patients from RTOG 0539 (Figs. 5D and 5E) and was well calibrated with 5-year PFS of 92.0%, 76.5%, and 38.6% for low, intermediate, and high risk groups, respectively. Moreover, the gene expression biomarker remained independently prognostic on multivariate analysis incorporating meningioma setting (primary or recurrent), extent of resection, and WHO grade using data from RTOG 0539 (Table S14).

## Discussion

Here we use targeted gene expression profiling to develop and validate a polygenic biomarker that provides additional information for meningioma outcomes compared to recent classification systems, including prediction of postoperative radiotherapy responses. The gene expression biomarker we report is independently prognostic across all clinical, histological, and molecular contexts tested^5,16,17,19,21-24^, including WHO 2021 grade^11^, the current standard of care. When incorporated into clinical risk groups defined by contemporary trials^7-9^ that are consistent with consensus NCCN and EANO guidelines^4,35^, the gene expression biomarker re-classifies up to 52.0% of meningiomas and potentially refines postoperative management for 29.8% of patients.

DNA methylation profiling^21-24,36^, CNV analysis^16-18^, DNA sequencing^12-15^, and RNA sequencing^18-20,22^ have improved understanding of meningioma biology. Unsupervised bioinformatic analyses paired with mechanistic and functional approaches have identified molecular groups and subgroups of meningiomas with distinct biologic drivers, therapeutic vulnerabilities, and clinical outcomes^18,19,21,22^. Supervised bioinformatic models incorporating clinical endpoints have refined risk stratification for meningioma local recurrence^16,17,24,36^. The gene expression biomarker reported here provides additional prognostic information for local recurrence and overall survival when combined with all unsupervised or supervised meningioma molecular classification systems tested. These findings are concordant with pan-cancer analyses examining gene expression, CNV, DNA methylation, protein expression, and DNA sequencing data in 10,884 patients, which suggest gene expression encodes the greatest prognostic information across cancer types^28^. In support of these data, targeted gene expression biomarkers and continuous risk scores have proven successful for multiple cancers^25-27,37-39^, particularly for breast cancer where polygenic biomarkers are standard of care^25,30^. Previous efforts to reduce meningioma molecular classification systems to immunohistochemical stains have thus far not been reproducible^40^. More broadly, qualitative or semi-quantitative protein expression is unlikely to capture the quantitative signal of a gene expression continuous risk score, especially when incorporating non-protein coding genes, as is the case for the biomarker we report (Fig. 1E, Table S3).

Current indications for postoperative radiotherapy for patients with meningiomas are controversial, particularly for patients with primary WHO grade 2 meningiomas who are randomized to postoperative surveillance or postoperative radiotherapy on NRG BN-003 and EORTC 1308 after GTR^3,6^. Conflicting retrospective series have variably reported a benefit^9,41-48^ or no benefit from radiotherapy in this setting^47,49-56^, which has fueled debate and inspired these international Phase III clinical trials of radiotherapy for patients with meningiomas. The gene expression biomarker reported here improves risk stratification for primary WHO grade 2 meningiomas and may identify favorable WHO grade 2 meningiomas where postoperative radiotherapy could be safely omitted in favor of close surveillance. The gene expression biomarker also identifies primary WHO grade 1 meningiomas with elevated risk of recurrence (Figure S8E). Indeed, 6.4% of primary WHO grade 1 meningiomas in the multicenter retrospective clinical validation cohort were classified as gene expression high risk (N = 27), with 5-year LFFR of 43.0%. Of these, only 1 patient received postoperative radiotherapy (3.7%). The gene expression biomarker also identified 59 primary WHO grade 1 meningiomas (13.9%) with subtotal resection in the multicenter retrospective clinical validation cohort as intermediate risk, and this unfavorable combination was associated with 5-year LFFR of 65.1%. Of these, only 3 patients (5.1%) received postoperative radiotherapy. In sum, 20.3% of primary WHO grade 1 meningiomas in the multicenter retrospective clinical validation cohort (N = 86 of 423) were re-classified as unfavorable using biomarker/surgical strata, and the overwhelming majority of these patients did not receive postoperative radiotherapy (95.3%). To address these missed opportunities for refined risk stratification, the performance characteristics, rate of re-classification, and rate of potential refinement of postoperative management offered by the gene expression biomarker reported here compare favorably to well-established biomarkers in routine clinical use for patients with other cancers^29,30,57,58^.

Previous meningioma molecular classification studies have largely not reported overall survival outcomes. A prospective trial of trabectedin in 90 patients with recurrent WHO grade 2 or grade 3 meningiomas examined DNA methylation families in multivariate analysis without including WHO grade as a covariate, and found meningiomas in the malignant DNA methylation family had worse overall survival compared to non-malignant families, although all families (including benign and intermediate) experienced poor outcomes^59^. The data we present using meningiomas from RTOG 0539 demonstrate the gene expression biomarker was prognostic for overall survival both before and after adjusting for WHO grade on multivariate analysis, and that outcomes remained well-calibrated in this prospective, investigator-blinded validation cohort. For patients with meningiomas, prospective trials such as these will be critical to distinguish conventionally higher risk cases that may safely undergo postoperative surveillance (Figures S8F, S8G), elucidate which biomarker(s) could be used for stratification (Figures S8H, S8I), and determine whether the timing of postoperative radiotherapy or other interventions improves overall survival (Figure S8J). As clinical trials develop, we do not anticipate targeted gene expression profiling will obviate longstanding and robust meningioma classification systems, such as WHO grade^11^, or more recent classification systems that are tractable across multiple brain tumor types, such DNA methylation profiling which elucidates biological drivers and vulnerabilities to molecular therapy for meningiomas^21,22,60^. Rather, if incorporated alongside other meningioma classification systems and clinical factors such as extent of resection that are already in widespread use, the gene expression biomarker reported here may offer additional benefit to patients with the most common primary intracranial tumor^1^, particularly in terms of postoperative radiotherapy response.

This study should be interpreted in the context of its limitations. First, clinical data in the discovery and multicenter validation cohorts were obtained retrospectively, suggesting our results are susceptible to biases inherent to retrospective research. To address this limitation, we provide additional investigator-blinded, independent validation using meningiomas and clinical data that were prospectively collected from patients enrolled on RTOG 0539. Second, pathology and radiology reviews were performed independently at each institution for meningiomas in the retrospective discovery and validation cohorts. Nevertheless, inter-observer concordance for meningioma WHO grade and imaging characteristics are high^61-63^, and any heterogeneity in clinical review across independent cohorts may better represent the heterogeneity intrinsic to routine clinical practice than might be anticipated from central review. To further address this limitation, the meningiomas from RTOG 0539 that were included in this study underwent central pathology and radiology review^7-9,63^.

## Conclusions

Targeted gene expression profiling of meningiomas identifies, optimizes, and validates a biomarker predicting local recurrence, overall survival, and radiotherapy benefit, re-classifying up to 52.0% of meningiomas compared to conventional clinical criteria and potentially refining postoperative management for 29.8% of patients.

## Figures and Tables

**Table 1.** Discovery and clinical validation cohort characteristics.

	Discovery	Retrospective clinical validation	Prospective clinical validation
**Meningiomas - no.**	173	866	103
**Patients - no.**	166	801	103
**Females - no. (%)**	112 (67.5)	543 (68.7)	68 (66.0)
**Median age (IQR) - yr.**	57.0 (45-65.1)	58.9 (48.6-67.6)	57 (49-65)
**Setting - no. (%)**			
Primary	143 (82.7)	635 (80.1)	81 (78.6)
Recurrent	30 (17.3)	153 (19.4)	22 (21.4)
Not available	0 (0.0)	78 (9.0)	0 (0.0)
**Extent of resection - no. (%)**			
Gross total	110 (63.6)	541 (69.8)	70 (68.0)
Subtotal	63 (36.4)	234 (30.2)	17 (16.5)
Not available	0 (0.0)	91 (10.5)	16 (15.5)*
**WHO grade - no. (%)** **			
1	83 (50.0)	499 (57.6)	51 (49.5)
2	65 (37.6)	240 (27.7)	37 (35.9)
3	25 (14.4)	127 (14.7)	15 (14.6)
**Gene expression risk score - no. (%)**			
Low	63 (36.4)	252 (29.1)	39 (37.9)
Intermediate	72 (41.6)	406 (46.9)	46 (44.7)
High	38 (22.0)	208 (24.1)	18 (17.5)
**Postoperative radiotherapy - no. (%)**	33 (19.1)	147 (17.3)	63 (61.1)
**Median follow up (IQR) - yr.**	8.1 (3.9-11.9)	5.2 (2.3-8.7)	8.4 (5.1-9.3)
**Local recurrence - no. (%)**	61 (35.3)	253 (29.2)	29 (28.2)***
**Death - no. (%)**	46 (26.6)	190 (21.9)	21 (20.4)

The discovery cohort was comprised of frozen meningiomas from a single institution (UCSF, Table S2). The non-overlapping retrospective clinical validation cohort was comprised of frozen (N=572) and FFPE meningiomas (N=294) from 6 institutions: consecutive meningiomas from The University of Hong Kong (Table S5), and non-consecutive meningiomas from Northwestern University (Table S6), UCSF (Table S7), Baylor College of Medicine (Table S8), Heidelberg University and Medical University of Vienna (Table S9). The non-overlapping prospective clinical validation cohort was comprised of FFPE meningiomas from RTOG 0539 (Table S13), a completed prospective clinical trial of postoperative radiotherapy versus postoperative observation for patients with meningiomas.

* Some recurrent meningiomas from patients enrolled on RTOG 0539 received radiotherapy without repeat surgery.

** WHO 2016 grade based on histological criteria.

*** The events from RTOG 0539 were defined as progression or death, and outcomes for this cohort are reported as progression free survival or overall survival.
